# Associations Between Defence-Style, Eating Disorder Symptoms, and Quality of Life in Community Sample of Women: A Longitudinal Exploratory Study

**DOI:** 10.3389/fpsyg.2021.671652

**Published:** 2021-07-01

**Authors:** Phillip Aouad, Phillipa Hay, Nasim Foroughi, Suzanne M. Cosh, Haider Mannan

**Affiliations:** ^1^InsideOut Institute, Faculty of Medicine, University of Sydney, Camperdown, NSW, Australia; ^2^School of Psychology, University of New England, Armidale, NSW, Australia; ^3^Translational Health Research Institute, Western Sydney University, Sydney, NSW, Australia

**Keywords:** defence-style, eating disorders, quality of life, disordered eating, women

## Abstract

**Background and Aim:** Eating Disorders (EDs) impact an estimated 15% of the global population and are linked to maladaptive defence-styles (coping strategies) and poorer mental health outcomes. Defence-styles have been grouped into immature, neurotic, and mature behaviours. Studies have yet to examine all three defence-styles in ED symptomatic individuals over an extended period of time. The current study aimed to investigate using converse analysis the relationships between defence-style and ED outcomes over a 5-years period.

**Methods:** Participants (*n* = 216, mean age 33 years) were recruited through the Women's Eating and Health Literacy study, with the current study examining a 5-years period of two waves (year-4 and year-9). The current study tested associations over time between eating pathology (EDE-Q), psychological distress (K10), mental and physical health related quality of life (M/PHRQoL, SF-12), and defence-style (DSQ-40).

**Results:** Mature, immature and neurotic defence-styles did not significantly change over 5 years. Over the same period, only PHRQoL significantly predicted mature defence-styles having positive effect. Both MHRQoL and PHRQoL significantly predicted immature defence-styles having positive and negative effects, respectively. Psychological distress, PHRQoL and weight concern significantly predicted neurotic defence-styles having positive effects except for psychological distress. PHRQoL, MHRQoL, restraint and eating concern significantly predicted overall eating pathology having positive effects except for PHRQoL and MHRQoL. Conversely, among the defence-style variables, over 5 years, both immature and neurotic defence-styles significantly predicted psychological distress having positive effects, immature and mature defence-styles significantly predicted MHRQoL having negative and positive effects, respectively, while only immature defence-styles significantly predicted overall eating pathology having positive effect.

**Conclusions:** The results of the current study suggest that immaturity and neuroticism but not maturity were the defence-style variables predicting psychological distress over a 5-years period while conversely psychological distress predicted only neurotic defence styles. The findings of the current study may suggest that without intervention, mature, immature and neurotic defence-styles may largely remain immutable to significant shifts over time. Limitations in the current study included limited demographic representation. The current study is anticipated to generate considerations into treatments that could strengthen defence-styles in individuals with increased eating pathology.

## Introduction

### Overview

The impact of an Eating Disorder (ED) on an individual's life can hinder their ability to cope with stressful situations (Ziegler, 2016). How a person copes with stressors (defence-style) in their environment is said to be a result of their subconscious mind, and can be altered by the presence of psychiatric illness (Vaillant, 1994). While research into the influence of eating disorders on defence-styles has been explored empirically, there appears to be a dearth in knowledge in determining if defence-styles influence eating disorders. If there is a relationship between eating disorders and defence-styles, it may stand to provide insight into potentially enhancing ED therapies and treatments to produce more effective outcomes. This paper is an exploratory study reporting on the relationship between EDs and defence-styles in a community sample of women.

### Defence-Styles

Defence-styles, or defence mechanisms, are coping strategies at varying levels of adaptive coping (Ziegler, 2016). Defence-styles are anchored in psychological processes that occur subconsciously in order to reduce negative emotional responses caused by undesirable stimuli (Steiger and Zanko, 1990). Defence-styles were first hypothesised by Freud (1894), and to date include those of displacement, intellectualisation, projection, denial, rationalisation, reaction formation, repression, regression and sublimation (Ziegler, 2016). American psychiatrist Vaillant (1994) reorganised Freud's defence-styles into varying levels of: pathological, mature, immature, and neurotic styles, which scholars extensively use as a theoretical framework in current research to postulate underlying mechanisms that may explain certain behaviours (see Table 1) (Cramer, 2000; Cheng et al., 2015; Sala et al., 2015). Broadly, immature defence-styles often centre on distancing or ignoring one's response to a negative stimulus; mature defence-styles are centred around actively redirecting emotions in response to a negative stimuli to more adaptive situations or interactions; and neurotic defence-styles focus on controlling the emotional response to a negative stimuli.

**Table 1:** Vaillant (1994) defined defence mechanisms.

**Pathological (level I)**	**Immature (Level II)**	**Neurotic (Level III)**	**Mature (Level IV)**
Psychotic denial Delusional projection	Fantasy Projection Passive aggression Acting out	Intellectualisation Reaction formation Dissociation Displacement Repression	Humour Sublimation Suppression Altruism Anticipation

*Different variations exist of Freud's defence-styles, however all often have the core groups of immature, neurotic, and mature defence-styles. In the DSQ pathological are grouped with immature*.

Healthy and unhealthy consequences may result to the individual, dependant on the frequency and circumstance the defence styles are used (Weiten, 2007; Costa and Brody, 2008). Psychoanalytic theory indicates that the subconscious mind can manipulate, deny or distort a person's perception of reality in order to protect against inappropriate impulses, anxieties, stimuli or emotions, and to maintain one's self-schema or other schema's (perceptions), an individual may have of the world (Bond et al., 1983; Steiger et al., 1989, 1990; Steiner, 1990; Schmidt et al., 1993; Peteet, 2001; Hart et al., 2011; Ziegler, 2016).

### Measuring Defence-Styles

The construct of defence-style can be difficult to measure; however, over the years there have been several tools developed to assess defence-styles (Laor et al., 2001). Laor et al. (2001) indicate that among the more commonly known measures are the: Defence Mechanism Inventory (Gleser and Ihilevich, 1969); Hierarchy of Defence Mechanisms (Vaillant, 1976); Defence Style Questionnaire (DSQ) (Bond et al., 1983); Defence Mechanism Rating Scale (Perry and Cooper, 1989); Defence Mechanism Manual (DMM) cram (Cramer and Blatt, 1990); and Comprehensive Assessment of Defence-Style (CADS) (Laor et al., 2001). However, given its brevity, simplicity, specificity, and strong validations the modified version of the DSQ, the DSQ-40, is perhaps one of the current and commonly used self-reported measures of defence-styles (Andrews et al., 1993).

### Current Understanding of the ED and Defence-Style Link

Bond and Perry (2004) conducted a study on defence-style relationships with various psychopathology and change in outcomes, and found that variations in the utilisation of defence-styles may be seen in particular patient groups with specific disorders. For example, anxiety and depression appear to both be positively associated with immature and neurotic defence-styles, but negatively associated with mature defence-styles (Spinhoven and Kooiman, 1997). Nonetheless, while adaptive defence-styles are often seen to improve with symptom reduction, the author argues that defence-styles may also be an indicator or even a predictor of the intervention (therapy) being provided to the patient (Bond, 2004).

Individuals with Eating Disorders (EDs) are often seen to utilise variants of these defences, which may contribute to their ED and the maintaining of disordered eating behaviours (Zeigler-Hill et al., 2008; Goulia et al., 2015). Alternatively, it may also be that ED symptoms have some impact on an individual's defence style (Gitzinger, 1993; Sullivan et al., 1994; Vidović et al., 2003). Nonetheless, research into such relationships between eating pathology and defence-styles has yet to be explored. Hay and colleagues (Hay et al., 2010) began to longitudinally examine the natural history and possible predictors, including defence-styles, of various outcomes in college-age females with common eating disorders. Participants who exhibited eating disorder symptoms appeared to score higher on immature and neurotic defence styles, and lower on mature defence styles (Hay and Williams, 2013). Specifically, participants who had higher baseline scores for immature and neurotic defence-styles had a higher level of ED symptomatology and poorer MHQoL at 2-years follow-up; when compared to participants who scored lower on baseline immature and neurotic defence styles (Hay et al., 2010).

In a continuation of the above study, Hay and Williams (2013) reported that at year-4 and year-5 follow-up participants with higher immature and neurotic defence-style scores continued to report higher levels of ED symptomology compared to community norms. Analysis using multivariate linear modelling showed that perceived stress, immature defence-style, and psychological distress were still significantly associated with ED symptoms at both year-4 and year-5 (Hay and Williams, 2013). According to Hay and Williams (Hay and Williams, 2013), women at year-5 follow-up continued to show signs of pathological eating significantly associated with immature defence-styles at baseline. Conversely, given that defence-styles are capable of influencing an individual's psyche, it may stand to reason that this may extend to an individual's psychopathology influencing their defence-style; but to our knowledge, no examination of this converse relationship with regard to ED symptoms and defence-style has been done. Investigating this relationship may offer other avenues of ED treatment, such as focusing on improving defence-style to improve eating pathology in individuals who may not respond to conventional treatments that target the maintaining behaviour (Fairburn et al., 2003). Moreover, although studies have found that mature and neurotic defence-styles are less variable over time than immature defence-styles, the opposite relationship between defence-style changes over time has also yet to be examined.

Therefore, the current study will aim to extend on Hay and Williams (2013) previous findings to see if these continue to be seen over an extended period of time (from baseline year-4 to follow-up at year-9). To address the limitations of Hay and Williams (2013) study, the current study will examine the opposite relationships in time in all three defence-styles with ED symptoms and will also investigate the predictors of defence-style changes overtime in relation to MHRQoL, as well as psychological distress. Based on previous research we anticipated that a more immature defence-style would be associated with higher ED symptoms overtime. The converse relationship between ED symptoms at baseline and defence-styles at a follow-up time period is exploratory and thus no hypotheses are made.

## Methodology

### Study Design and Procedure

The present study was nested in The Women's Eating and Health Literacy longitudinal study (hereafter WEHL), with initial ethics approval granted by James Cook University, and subsequent approvals/reviews approved by the Western Sydney University (WSU) Human Research and Ethics Committee (HREC; approval: H9283). Written informed consent was obtained from all participants prior to the commencement of the current study. Data were collected over 9-years in six waves (baseline, year-1, year-2, year-4, year-5, and year-9), to date. The WEHL study used pooled data from two cohorts that purposively oversampled for adult women with high levels of ED symptoms. The first cohort were ED symptomatic participants who were initially recruited from the general population of women aged 18–42 in the Australian Capital Territory (ACT), Australia (Mond et al., 2004; Mitchison et al., 2013). The second cohort were women aged over 18 years who were recruited from various Universities and Technical and Further Education (TAFE) Institutes across Queensland and Victoria, Australia. The cohorts were recruited over 24 months. The current study examined year-4 (Time 1; T1) and year-9 (Time 2, T2) data only.

For additional information on the larger study please see Mitchison et al. (2015) and Holtzhausen et al. (2020).

#### Mail-Out and Email Surveys

Invited individuals who preferred email contact were emailed electronic versions of the study and others sent paper copies by post. In order to ensure maximum response rate, surveys were sent out to non-responders at two, three and 4 months for both aforementioned cohorts (Mitchison et al., 2013, 2015). This procedure was repeated at each follow-up time-point.

### Participants

Of 828 baseline participants, 434 (52.4%) completed T1 follow-up, and 364 (44.0%) completed T2 follow-up. The mean age of the sample was 32.47 years old (*S.E* = 0.86) at T1. Over half (51.41%) of the sample indicated they had studied an undergraduate level bachelor's degree or higher at T1, with 77.7% indicating they had attained a bachelor's degree or higher at T2. Moreover, 35% of females indicated having a child/ren at T1, with an increase to 45% of participants indicating they had a child/ren at T2. Of the sample, 50.71% indicated being married at T1, which dropped to 32.4% at T2. The average time-out of their regular role (study or work) at T1 and T2 remained unchanged, with the average being three days for reasons unspecified (Media*n* = 1.00). Mean BMI at T1 was 26.09 kg/m^2^, which increased to 27.22 kg/m^2^ at T2. Additional sociodemographic characteristics and information can be found in Supplementary Tables 1, 2.

### Measures

To determine the demographic characteristics of the participants and their change-over time, the same questions relating to employment status, highest education, marital status, days out of their regular role (e.g., work or study), as well as self-reported height and weight were asked at each testing interval. Further to this, several measures were administered to determine eating pathology, psychological distress at the time of the study, and both Mental and Physical HRQoL components.

For the baseline demographic characteristic year four age there were no dropouts at year nine and were therefore unable to test whether mean year four age differed significantly between dropouts and study completers. However, for year four employment status there were some dropouts (*n* = 110) at year nine, using this variable it was noted that there was a significant difference between dropouts and study completers. Specifically, there was non-significant difference between year nine dropouts and completers for year four employment status (*p* = 0.6405 for Fisher's exact test), year four education (*p* = 0.5894 for Fisher's exact test), year four marital status (*p* = 1.00 for Fisher's exact test), and median days out of role (Z statistic = −0.5836, *p* = 0.5595 for Wilcoxon sum rank test). In summary, no differences were found between dropouts and completers for the baseline demographic characteristics.

### Eating Pathology (EDE-Q)

To assess eating disorder symptomology the Eating Disorder Examination Questionnaire (EDE-Q) was used (Fairburn et al., 2008; Mond et al., 2014). The EDE-Q measures eating disorder pathology (behaviour) in the preceding 28-days period and instructs participants to rate their severity and frequency of weight and shape concerns using a seven-point Likert scale (0 = No days/Not at all to 6 = Everyday/Markedly). The EDE-Q has four quantifiable subscales: (1) Weight Concern—a measure of the amount of worry an individual has about their weight; (2) Shape Concern—determines the impact of worrying about one's body figure (shape); (3) Eating Concern—the amount of anxiety surrounding eating; and (4) Restraint—a measure of how avoidant an individual is around food (Fairburn et al., 2008). Global (overall) eating pathology is calculated as a mean of the combined subscale scores, with higher global scores being more indicative of disturbed eating pathology (Fairburn et al., 2008). Global scale scores ≥2.3 together with any objective binge eating occurrences or the use of exercise for weight control purposes, is suggestive of ED pathology. Furthermore, the EDE-Q has good internal reliability across subscales: restraint (α = 0.82, five-items), eating concern (α = 0.86; five-items), shape concern (α = 0.92; eight-items), weight concern (α = 0.84; five-items) (Rose et al., 2013).

#### Defence-Style (DSQ)

The Defence-Style Questionnaire (DSQ-40) was used to assess defence-styles of participants (Andrews et al., 1993). This is a 40-item measure using a nine-point Likert response scale ranging from “strongly agree” to “strongly disagree,” and derives defence-style subscale scores by assessing 20 defence-mechanisms (Andrews et al., 1993). It should be noted that defence-styles and defence-mechanisms are different. Defence-mechanisms may be considered as individual behaviours as opposed to defence-styles, which may be thought of as a collection of behaviours in response to particular stimuli or events (Andrews et al., 1993). Defence-mechanisms are organised into three subscales (defence-styles): Mature (eight-items), Neurotic (eight-items), and Immature (24-items). Scores for defence-styles are calculated using the mean ratings for relevant items. Higher scores for a particular subscale indicate higher use of that particular defence-style in response to stimuli. The DSQ had good reliability across each of the three subscales for the current study; mature (α = 0.70), neurotic (α = 0.61), and immature (α = 0.83) defence-styles and the scale has been very well-validated across multiple cohorts (Andrews et al., 1993).

#### Psychological Distress (K10)

Psychological Distress, was measured using the Kessler Psychological Distress Scale (K10) (Andrews and Slade, 2001; Kessler et al., 2002). This scale was selected due to the brevity of administration time, simplicity of questions asked, and the ability of the K-10 to discriminate between clinical and non-clinical cases of psychological distress (Mitchison et al., 2013). Items used a 5-point Likert scale (ranging from 1. “None of the Time” to 5. “All of the Time”), and items were summed to provide a total score out of 50—where the higher the score, the higher the occurrence of psychological symptomatology. The K10 had very good internal consistency (α = 0.87), and has been validated for both individual and populous use (Kessler et al., 2002).

#### Health Related Quality of Life (SF-12)

Mental Health Related Quality of Life (MHRQoL) and Physical Health Related Quality of Life (PHRQoL) were assessed using the Short-Form-12 (SF-12) (Ware et al., 1996). The SF-12 assesses multiple dimensions of HRQoL including physical functioning, physical role, body pain, general health, vitality, social functioning, emotional role, and mental health—grouping these into two composite scores: Physical Composite Score (PCS) and Mental Composite Score (MCS) in order to provide the M/PHRQoL scores. Each of the domains is scored from 0 to 100, with higher scores indicating better QoL. The SF-12 had good reliability for both PCS (Cronbach's α = 0.87) and MCS (Cronbach's α = 0.77), and has been well-validated (Ware et al., 1996).

### Data Analysis

SAS 9.4 (SAS Version 9.4, 2013) was used for all data analyses except for delta method for which the R package ‘msm' (Jackson, 2011) was used. Datasets from year-4 (T1) and year-9 (T2) were combined, and duplicate cases (where responses were matched for T1 and T2) were combined or removed entirely if no data were entered for the duplicate entry. Prior to analysis, data were cleaned and checked in order to ascertain that all assumptions had been met for the chosen statistical test. Where necessary, adjustments in analysis (specifically: using non-parametric, Spearman's rank correlation, analysis equivalents for testing associations when normality is violated) were made accordingly. The *p-*values were estimated using two-sided tests. Data transformations were performed for conducting multiple linear regression when necessary. When the outcome was positively skewed, square-root transformation was used while for negatively skewed outcome, log_10_ [max(variable+1)-variable] transformation was used.

Further examination, after transformation, did not appear to indicate that data further violated the required assumptions. To find the regression results for the original untransformed variables, the estimates for regression coefficients and 95% CIs were back-transformed while to find the SEs the delta method was used. As indicated in the aims, data analysis was largely exploratory in nature, requiring systematic progression of the analytical techniques used, which began by examining change in defence-style over time, followed by examining the associations between variables, and finally analysing the predictors of psychological distress, PHQoL, MHQoL and overall eating pathology, and of the three defence-styles.

To determine the overall (mean) change over time of defence-styles, paired samples *t*-tests were conducted for each defence-style DSQ score (mature, neurotic, and immature; with normal distribution) differences over time (T2–T1). In these analyses all variables followed normal distribution. The corresponding Cohen's d statistic for a paired t-statistic was calculated to measure effect size for the change over time. It was then classified based on magnitude (Cohen, 1988). Associations were examined overtime between each of the defence-styles (mature, neurotic, and immature) and eating pathology (including subscales and global score), psychological distress, and M/PHRQoL Spearman rho (*r*_*s*_) analyses were used, due to non-normal distribution of some variables (including T1 psychological distress, and T1 and T2 PHRQoL). A series of multiple linear regressions (MLR) were conducted to examine the T1 predictors of psychological distress, PHQoL, MHQoL and overall eating pathology measured at T2 while controlling for demographic features at T1. A series of MLRs were also conducted to examine the T1 predictors of the three defence-style variables measured at T2, while controlling for demographic features, at T1. Using MLR allowed examination of between subject differences in defence-styles, and allowed for the assessment of each predictor's influence to the overall variance in DSQ subscale scores between each of the two time points. We also fitted MLRs with the dependent variable score at T1 as a covariate in the model plus the sociodemographic variables and psychological variables as predictors. The dependent variable (DV) for these models is mature defence style, immature defence style, neurotic defence style and overall eating pathology, respectively. Year 4 age was the only demographic variable entered in the models because it was found to be a confounder (operational) for most of the predictors included in the models. It was not found to be a significant predictor of the DVs in some of the models. An operational confounder does not require to be a significant predictor of the DV to be included in the model. Note that the operational definition of confounding provides a stronger adjustment of confounding than the classical definition (Mamdani et al., 2005). Because year 4 age is a confounder it was entered as a control variable in each model and hence the regression results of this variable are not reported. All missing data at baseline (year 4) were multiply imputed using multivariate normal imputation. There were 25 imputations performed. There were two auxiliary variables used in the imputation models: year 4 BMI and year 4 age. The results for multiple imputation were pooled using Rubin's method (Rubin, 1987). Multicollinearity for all regression analyses was tested using variance inflation factor (VIF) and tolerance values. No multicollinearity was detected for any of the predictors as none of the VIF values were above 10 or none of the tolerance values were above 0.1.

Reported effect sizes (Cohen's *d*) are based on cut-off criteria as specified by Cohen (1988). Effects have been classified in terms of magnitude with a cut-off of *d* = 0.2 considered a ‘small' effect size; a cut-off of *d* = 0.5 considered a “medium”; and a cut-off of *d* = 0.8 considered a ‘large' effect size (Cohen, 1988).

## Results

### Cross-Sectional Associations Between Defence-Styles, HRQoL, Psychological Distress, and Eating Pathology

At T1, there was a significant positive association between an immature defence-style and overall eating pathology, *r*_s_ = 0.89, *p* < 0.001, and a weak significant association between overall eating pathology and mature defence-style *r*_s_ = −0.13, *p* < 0.005. There were no significant associations between mature or neurotic defence-style and overall eating pathology. All other within T1 associations are presented in Supplementary Table 3.

### Defence-Style Changes Over Time

Results of a paired samples *t*-tests between T2 immature defence styles (*Mean* = 3.46, *S.E* = 0.04) and T1 immature defence-style (*Mean* = 3.44, *S.E* = 0.94) indicated a non-significant reduction in scores over time between scores; *t*_(606)_ = −1.36, *p* = 0.173. The effect size for change in immature defence style over time is −0.039 indicating weak (Cohen, 1988) negative effect. Neither neurotic defence styles (*Mean* = 4.58, *S.E* = 0.05; T2) nor mature defence-styles (*Mean*=5.37, *S.E* =0.05; T2) indicated significant changes over time [*t*_(606)_ = −1.48, *p* = 0.140; *Mean*_yr4_ = 4.58; *S.E*_yr4_ = 0.062; neurotic T1] [*t*_(606)_ = −0.87, *p* = 0.382; *Mean*_yr4_ = 0.082; *S.E*_yr4_ = 0.132; mature defences T1]. Cohen's d for these changes are −0.0425 and −0.025, respectively. Both these effects are small (Mitchison et al., 2013).

Supplementary Table 4 presents between all T1-and-T2 associations among defence-styles and other assessed psychometric measures.

### Impacts of T1 Eating Pathology, Weight, Eating, and Shape Concerns, Restraint, HRQoL & Psychological Distress on T2 Defence-Styles

MLRs were fitted to predict each of the T2 defence-styles based on T1 eating pathology (weight concern, eating concern, shape concern, restraint and overall), MHQoL, PHQoL, and psychological distress. Overall F test was statistically significant in all models (Table 2A). The *R*^2^ values ranged from 0.039 to 0.403 and adjusted *R*^2^ ranged from 0.028 to 0.396, for these models (Table 2A).

**TABLE 2A:** Overall F test and fit statistics of the models with eating pathology subscales, quality of life & demographic factors predicting defence-styles.

**Dependent variable**	**Model df**	**Error df**	**F-value**	***p-*value**	***R*^**2**^**	**Adj-*R*^**2**^**
Immature defence-style	8	598	3.38	<0.001	0.043	0.032
Mature defence-style	8	598	2.99	<0.010	0.039	0.028
Neurotic defence-style	8	598	4.55	<0.0001	0.057	0.046
Overall eating pathology	8	598	4.55	<0.0001	0.403	0.396

Partial regression coefficients (B), and squared partial correlations (*sr*^2^) for each predictor used in the regression models are reported in Table 2A while overall *R*^2^ values are reported in Table 2B. These results are discussed below for each model.

**TABLE 2B:** Influence of psychological distress, HRQoL, and eating pathology at T1 on defence-styles at T2.

**Variables (T1)**	**B**	**SE**	**95% CI**	**sr^**2**^**	***P-value***
**Dependent variable is mature at T2**					
Psychological distress	−0.010	0.008	−0.025, 0.005	0.015	0.21179
PHQoL^**^	0.022	0.006	0.009, 0.034	0.020	0.00027
MHQoL	0.003	0.005	−0.006, −0.001	0.010	0.54873
Weight concern	−0.008	0.057	−0.120, 0.105	0.001	0.88843
Eating concern	0.003	0.057	−0.110, 0.116	0.000	0.95804
Shape concern	−0.018	0.057	−0.131, 0.095	0.000	0.75227
Restraint	−0.016	0.040	−0.095, 0.062	0.000	0.68930
**Dependent variable is immature at T2**					
Psychological distress	−0.001	0.006	−0.013, 0.010	0.001	0.86769
PHQoL^*^	0.013	0.005	0.004, 0.023	0.010	0.00955
MHQoL^*^	−0.008	0.004	−0.015, 0.008	0.003	0.04595
Weight concern	0.039	0.044	−0.047, 0.124	0.008	0.37578
Eating concern	−0.006	0.044	−0.092, 0.080	0.000	0.89158
Shape concern	0.066	0.044	−0.020, 0.152	0.003	0.13414
Restraint	−0.053	0.081	−0.113, 0.006	0.005	0.51316
**Dependent variable is neurotic at T2**					
Psychological distress^*^	−0.014^*^	0.007	−0.028, −0.000	0.001	0.04595
PHQoL^*^	0.018^*^	0.006	0.006, 0.030	0.020	0.00281
MHQoL	−0.010	0.009	−0.018, 0.001	0.012	0.26697
Weight concern^*^	0.135^*^	0.054	0.030, 0.241	0.027	0.01269
Eating concern	−0.044	0.054	−0.151, 0.062	0.001	0.41550
Shape concern	0.041	0.054	−0.065, 0.148	0.001	0.44800
Restraint	−0.021	0.038	−0.095, 0.053	0.000	0.58072
**Dependent variable is overall eating pathology at T2**					
Psychological distress	−0.001	0.013	−0.04, 0.02	0.116	0.93871
PHQoL^**^	−0.031^**^	0.007	−0.05, −0.02	0.028	0.00011
MHQoL^**^	−0.040^**^	0.006	−0.05, −0.03	0.106	<0.00001
Weight concern	0.140	0.094	−0.046, 0.325	0.131	0.13692
Eating concern^*^	0.173^*^	0.081	0.013, 0.332	0.031	0.03310
Shape concern	0.094	0.094	−0.092, 0.281	0.380	0.31771
Restraint^**^	0.186^**^	0.055	0.082, 0.291	0.005	0.00077
Immaturity	0.085	0.111	−0.139, 0.309	0.003	0.44412

* *Significant to p < 0.05*;

** *significant to p < 0.001*.

*All models controlled for year 4 age*.

**Mature defence-style**. In combination, T1 psychological distress, weight concern, eating concern, shape concern, restraint, MHQoL, PHQoL, overall eating pathology and age accounted for 8.6% of the variance in T2 mature defence-style (*R*^2^ = 0.086), however except for PHQoL which had a negative significant effect (*p* < 0.001), none of these factors were individually significantly associated with mature-style.

**Neurotic defence-style**. In combination, T1 psychological distress, weight concern, eating concern, shape concern, restraint, MHQoL, PHQoL, overall eating pathology and age accounted for 5.0% of the variance in T2 neurotic defence style, with three of these factors, psychological distress, PHQoL and weight concern individually significantly associated with neurotic defence-style. T1 PHQoL and weight concern increased T2 neurotic defence style while MHQoL decreased it.

**Immature defence-style**. In combination, T1 psychological distress, MHQoL, PHQoL, restraint, overall eating pathology, weight concern, eating concern, shape concern and age accounted for 8.0% (*R*^2^ = 0.08) of the total variance observed in the model. A significant regression coefficient (*p* < 0.05) was noted for T1 MHQoL and PHQoL but not for T1 psychological distress restraint, eating pathology, weight concer, eating concern and shape concern. T1 PHQoL increased T2 immature defence style while T1 MHQoL decreased it.

**Eating pathology**. In combination, T1 immaturity, psychological distress, MHQoL, PHQoL, restraint, weight concern, eating concern, shape concern and age accounted for 40.3% (*R*^2^ = 0.403) of the total variance observed in the model. A significant regression coefficient (*p* < 0.05) was noted for T1 MHQoL, PHQoL, eating concern and restraint but not for T1 psychological distress, weight concern and shape concern. T1 MHQoL and PHQoL reduced T2 overall eating pathology while T1 eating concern and restraint increased it.

Supplementary Table 5 presents regression results for all three defence-styles and overall eating pathology and T1 predictors.

### Impact of T1 Defence-Styles on T2 Psychological Distress, PHQoL, MHQoL, and Overall Eating Pathology

Multiple linear regressions were fitted to predict T2 psychological distress, PHQoL, MHQoL, and overall eating pathology based on T1 defence-styles. Overall F test was statistically significant for all models (Table 3A).

**TABLE 3A:** Overall F test and fit statistics of the models with defence style and demographic factors predicting psychological distress, PHQoL, MHQoL, and overall eating pathology.

**Dependent variable**	**Model df**	**Error df**	**F Value**	***p*-value**	***R*^**2**^**	**Adj *R*^**2**^**
Psychological distress	4	602	32.65	<0.0001	0.178	0.173
PHQoL	4	602	9.60	<0.0001	0.060	0.054
MHQoL	4	602	22.74	<0.0001	0.131	0.125
Overall eating pathology	4	602	75.64	<0.0001	0.059	0.053

Partial regression coefficients (B), and squared partial correlations (*sr*^2^) for each predictor used in the regression models are reported in Table 3A while overall R^2^ values are reported in Table 3B. These results are discussed below for each model.

**TABLE 3B:** Influence of defence-style factors at T1 on T2 psychological distress, PHQoL, MHQoL, and overall eating pathology.

**Variable**	**B**	**SE**	**95% CI**	**sr^**2**^**	***P-value***
**Psychological distress T2**					
Immature T1^**^	2.860	0.488	1.881, 3.835	0.068	<0.00001
Mature T1	−0.109	0.348	−1.911, 0.529	0.009	0.75422
Neurotic T1^*^	0.856	0.385	0.261, 1.270	0.009	0.02656
**PHQoL T2**
Immature T1	−0.916	0.552	−2.013, 0.181	0.008	0.09395
Mature T1	0.014	0.365	−0.828, 0.610	0.003	0.96942
Neurotic T1	−0.236	0.476	−1.183, 0.711	0.005	0.62022
**MHQoLT2**					
Immature T1^**^	−2.520^**^	0.564	−0.363, −1.412	0.064	<0.00001
Mature T1^**^	1.785^**^	0.506	0.783, 2.787	0.029	0.00045
Neurotic T1	−1.125^*^	0.571	−2.258, −0.008	0.011	0.04927
**Overall eating pathology T2**					
Immature T1^*^	0.254	0.090	0.072, 0.436	0.048	0.00493
Mature T1	−0.127	0.075	−0.276, 0.022	0.004	0.09091
Neurotic T1	−0.076	0.080	−0.083, 0.235	0.004	0.34249

* *Significant to p < 0.05*;

** *significant to p < 0.001, All models controlled for year 4 age*.

**Psychological distress**. In combination, T1 immature, mature and neurotic defence style variables and age accounted for 17.8% (*R*^2^ = 0.178) of the total variance observed in the model. T1 immature-defence style significantly (*p* < 0.001) decreased psychological distress at T2 accounting for 6.8% variance (see Table 3B). Neurotic defence style also significantly (*p* < 0.05) increased psychological distress score but T1 mature defence style was not a significant predictor of psychological distress.

**Mental health related quality of life**. In combination, T1 immature, mature and neurotic defence style variables and age accounted for 13.1% (*R*^2^ = 0.131) of the total variance observed in the model. T1 immature and mature-defence styles significantly decreased and increased MHQoL, accounting for 6.4 and 2.9% variance, respectively. Neurotic defence style was not a significant predictor of MHQoL.

**Physical health related quality of life**. In combination, T1 immature, mature and neurotic defence style variables and age accounted for 6% (*R*^2^ = 0.06) of the total variance observed in the model. Neither T1 mature nor immature defence style nor neurotic defence style was a significant predictor of PHQoL.

**Overall eating pathology**. In combination, T1 immature, mature and neurotic defence style variables and age accounted for 5.9% (*R*^2^ = 0.059) of the total variance observed in the model. Neither T1 mature defence style nor neurotic defence style was a significant predictor of overall eating pathology. T1 immature-defence style significantly (*p* < 0.05) increased overall eating pathology.

## Discussion

The first finding of the current study was a significant positive association between an immature defence-style and overall eating pathology, and a weak significant association between overall eating pathology and mature defence-style. No associations were found between eating pathology and neurotic defence-styles. This is consistent with Bond and Perry's (2004) conclusions that variations in defence-styles are to be expected among different patient groups with varying psychopathologies. Second, no defence-style changed significantly overtime; potentially indicating that defence-styles as a whole may be relatively immutable. However, immature defence-styles showed a non-significant decrease over time with a weak negative effect. When examined together, T1 psychological distress, weight concern, eating concern, shape concern, restraint, MHQoL, PHQoL, overall eating pathology and age accounted for only 8.6 and 5.0% of the change in mature and neurotic defence-style, respecitively, at T2. This would suggest that other factors may influence how much defence-styles (specifically mature and neurotic defence-styles) may change overtime. As highlighted further below, and as highlighted in Bond and Perry's study (Bond and Perry, 2004), the presence of therapy may influence the variance seen overtime between defence-styles and a range of psychopathologies.

Further to the above, defence-styles were relatively stable between T1 and T2 (year-9), except for mature defence-styles scores, which increased on average by 5.29 (*M*_*diff*_ = 0.08–5.37). However, no differences reached statistical significance. Outside of the eating disorder context significant changes in defence-style overtime (particularly in mature and immature defence-styles) have been reported in the presence of therapy or treatment (Akkerman et al., 1999; Mullen et al., 1999; Cramer, 2007; Schauenburg et al., 2007; Hill et al., 2015). In the absence of intervention for particular psychological disorders, it has been found that neurotic and immature defence-styles reduced significantly while mature defences remained relatively stable, or increased marginally, over a 5–10 years period in late adolescents to early adulthood (Tuulio-Henriksson et al., 1997). The mean age of participants in the current study was 33 years old, and it is possible that in this older age group the defence style may be less likely to change.

Additionally, the findings of this study corroborate the findings by Hay and colleagues (Hay et al., 2010) and by Hay and Williams (Hay and Williams, 2013) who found that from baseline, immature defence-styles were associated with increased eating pathology, at year-2 and year-5 follow–ups, respectively. Moreover, the findings indicated that initial or early (T1) defence-style may predict to some extent, changes in overall eating pathology and MHQoL overtime, however these factors alone did not seem to significantly predict how mature-defence style would change over time. The findings therefore suggest that additional factors may contribute to the change in defence-styles over time. Such factors may include life experiences, environmental, and other physiological elements, which future studies should aim to address.

### Contextualisation of the Findings

Mental disorders, as a whole, can impede adaptive defence-styles. The more complex in nature the disorder, the more maladaptive defence-styles may present. Perhaps one of the most complex mental disorders, due to associated comorbidities, is eating disorders (Baucom et al., 2017). As previously alluded to in the introduction, psychodynamic theory relates eating disorders to inherent deficits in defence style including interpersonal functioning and self-regulation, and contends that abnormal eating pathology may attempt to reconcile tension regulation, an individual's sense of control, and the expression of interpersonal conflicts (Steiger and Houle, 1991).

Steiger and Houle (1991) found individuals with symptomatic eating disorder behaviours were more likely to exhibit maladaptive defence-styles. Moreover, the same study suggested that deficits in interpersonal and adaptive functioning may be antecedents to (as opposed to consequences of) the development of eating disorders (Steiger and Houle, 1991). Both Akkerman et al. (1999) and Bond and Perry (2004) assert that with psychodynamic therapy, long-term (>5 years) improvements in coping strategies and defence-styles can improve outcomes, particularly in individuals with depression—so far, no such study has been identified in the field of eating disorders. However, given the comorbidities often associated with eating disorders (depression, anxiety, obsessive compulsive tendencies, and trauma) (Hudson et al., 2007; Mond et al., 2009; Pini et al., 2013), defence-styles may improve more readily over an extended period of time in the presence of targeted ED-defence-style focused therapy.

### Strengths and Limitations

Strengths of the study include examining multiple facets of eating pathology and use of non-clinical cohorts allowed the current study to go beyond previous studies examining similar variables, which relied on clinical samples. Having a more representative sample, allows for results to potentially be more representative from the population they were drawn and allows for more widely applicable inferences to be made (Mitchison et al., 2013).

Limitations include the use of convenience sampling for one cohort through select educational institutions and those who had registered for prior research may have influenced outcomes, limiting generalisability. A further limitation relates to the measure used, where the internal consistency of the DSQ neurotic subscale (α = 0.61) was lower than both the mature (α = 0.70) and immature (α = 0.83) DSQ subscales. According to Clark and Watson (1995), the average recommended inter-item correlation should be between 0.15 and 0.50, but can vary based on the construct being measured. However, in the interest of transparency, the wider gap between subscale scores may indicate that an alpha score of 0.61, may be comparatively low to the other subscales, which may have potentially influenced results (Clark and Watson, 1995). Additionally, attrition (necessitating data imputation) and non-inclusions of males, who are severely underrepresented especially in eating disorder research (Striegel et al., 2012; Murray et al., 2017), may have further influenced findings. Lastly, culture and ethnicity were not examined in the sample which previous studies have indicated may influence ED and mental health outcomes, as well as defence-style development over time (Watson and Sinha, 1998; Walker and Lim, 2010; Foroughi et al., 2019). Future examinations of eating disorders and defence-styles should account and control for cultural and ethnic differences in participants.

### Clinical Significance

The current study is the first to examine longitudinal association of eating pathology and defence-styles in a community cohort. While this area of study still requires considerable research, the current study may offer academics and clinicians a starting point to consider treatments that may help increase adaptive defence-styles in order to reduce eating pathology over time, with treatments potentially focusing on transitioning individuals from using maladaptive defence-styles to adaptive styles, potentially lending itself to quicker recovery from an eating disorder. Specifically, treatments may focus on strengthening defence-styles by reducing immature defences and increasing mature defence-styles through therapy. Some efficacy for Cognitive Behavioural Therapy (CBT) has been found to influence defence-styles over time (Muris and Merckelbach, 1996; Coleman and Casey, 2007; Campbell et al., 2009). The findings of the current study suggest that more focus should be dedicated to investigating effective ways to instigate and maintain defence-style changes in individuals with eating disorder symptoms. This may be especially important in women with eating disorders, as previous studies (Evans et al., 2011; Hart et al., 2011) have shown that immature or maladaptive defence-styles may be a barrier to help-seeking in this population. There is some evidence to suggest that by increasing mature defence-styles individuals may begin to acknowledge, or accept, that they require professional help and may therefore be more likely to seek out treatment (Meyer, 2005).

### Future Directions

Future studies should focus on investigation of treatments that are best suited to influence defence-style change over time in eating disorder symptomatic individuals. Further, strengthening defence styles appears important, as mature defence-styles were associated with more positive eating disorder outcomes, compared to immature and neurotic defence-styles which were associated with more negative outcomes. Finally, research into which defence-styles are able to be targeted earlier rather than later, would be valuable to understanding if there is potential to improve patient outcomes from a younger age.

## Conclusion

The current study provides insight into the impact of increased eating pathology on shifts in defence-styles over-time. Overall, the study found that in adult women there was little change in defence style over time. Furthermore, the study highlights that therapeutic approaches that target shifting defence-styles in order to reduce eating pathology may be an avenue for treatment and research focus.

## Data Availability Statement

The raw data supporting the conclusions of this article will be made available by the authors, without undue reservation.

## Ethics Statement

The studies involving human participants were reviewed and approved by Human Research Ethics Committee Western Sydney University. The patients/participants provided their written informed consent to participate in this study.

## Author's Note

Part of the current research formed part of a thesis project conducted by PA–therefore some of the text utilised may be the same or similar to unpublished, published, or examined works written by PA.

## Author Contributions

PA conducted the literature review, data curation and data analysis, compiled the first draft, and undertook subsequent edits. PH undertook a supervisory role, data curation and data analysis, contributed to the first draft, and assisted with subsequent edits. NF conducted data curation and data analysis, contributed to the first draft, and assisted with subsequent edits. SC undertook a supervisory role, contributed to the first draft, and assisted with subsequent edits. HM conducted higher level data curation and data analysis, contributed to the first draft, assisted with subsequent edits, and took on an overall supervisory role/ senior academic. All authors contributed to the article and approved the submitted version.

## Conflict of Interest

PH received royalties from Hogrefe, and Huber and McGraw-Hill publishers for contributions on eating disorders. PH received royalties from Oxford University Press and receives sessional fees and lecture fees from the Australian Medical Council, Therapeutic Guidelines publication, and New South Wales Institute of Psychiatry. PH was a member of the World Health Organisation Working Group on Feeding and Eating Disorders for the Revision of ICD-10 Mental and Behavioural Disorders and this paper represents personal views of the author. PH has received an honorarium from Shire (now Takeda) Pharmaceuticals for a commissioned report and is an advisor to Takeda. The remaining authors declare that the research was conducted in the absence of any commercial or financial relationships that could be construed as a potential conflict of interest.
