# Supplementary material for: Associations Between Defence-Style, Eating Disorder Symptoms, and Quality of Life in Community Sample of Women: A Longitudinal Exploratory Study
Source: Front Psychol. 2021 Jul 1;12:671652. doi: 10.3389/fpsyg.2021.671652 (PMC8281956; doi:10.3389/fpsyg.2021.671652)
Supplement: Supplementary file 1 [file Table_1.docx]

**Supplementary Tables**

**Table S1**

***Frequencies of year 4 highest education obtained, year 4 work status, and year 4 marital status***

| **Highest Level Education** | **n** | **%** |
| --- | --- | --- |
| Year 10 | 13 | 3.67 |
| Year 12 | 61 | 17.23 |
| Trade Certificate/ Apprenticeship | 10 | 2.82 |
| Undergraduate Diploma | 23 | 6.50 |
| Bachelor’s Degree | 182 | 51.41 |
| Postgraduate Diploma | 30 | 8.47 |
| Post Graduate Degree | 35 | 9.89 |
| **Work Status** | **n** | **%** |
| Currently studying | 59 | 16.67 |
| Employed full time | 191 | 53.95 |
| Employed part-time/casually | 55 | 15.54 |
| Full time home duties/caring for children | 15 | 4.24 |
| Not employed- seeking employment | 6 | 1.69 |
| Not employed- not seeking employment | 6 | 1.69 |
| recovering from illness or injury | 9 | 2.54 |
| Other | 13 | 3.67 |
| **Marital Status** | **n** | **%** |
| Currently studying | 179 | 16.67 |
| Other | 174 | 3.67 |

***Note:*** *marital status total n=353; highest education total n=354; and work status total n=354*

**Table S2**

***Summary statistics of year 4 age and year 4 days out of role***

| **Sociodemographic Characteristic** | **Mean** | **Median** | **Range** | **S.E** | **IQR** |
| --- | --- | --- | --- | --- | --- |
| Age | 32.47 | - | 23-45 | 0.86 | - |
| Days out of role | - | 1.00 | 0-28 | - | 3.00 |

*Note:* For ‘days out of role’ median and IQR are reported as measures of central tendency and dispersion. This is due to the slightly skewed distribution of age

Table S3

*Summary of Intercorrelations (Spearman’s rho), Means, and Standard Errors between Defence-Styles subscales, Psychological Distress, HRQoL, & Eating Pathology (including subscales)*

|  |  |  |  | **Time 1 (Year 4)** | | | | | | | | | | |
| --- | --- | --- | --- | --- | --- | --- | --- | --- | --- | --- | --- | --- | --- | --- |
|  | ***Measures*** | **Mean** | **S.E** | **1** | **2** | **3** | **4** | **5** | **6** | **7** | **8** | **9** | **10** | **11** |
| **Time 1 (Year 4)** | **1. Distress** | \| 18.3 \| \| --- \| | \| 0.418 \| \| --- \| | - | -.01 | -.73^**^ | .50^**^ | .59^**^ | .55^**^ | .32^**^ | .91^**^ | -.19^**^ | .16^*^ | .49^**^ |
|  | **2. PHQoL** | 50.57 | 0.403 |  | - | -.27^**^ | -.09 | -.04 | -.10 | .10 | -.02 | -.06 | .03 | -.02 |
|  | **3. MHQoL** | 44.33 | 0.515 |  |  | - | -.37^**^ | -.41^**^ | -.38^**^ | -.27^**^ | -.37^**^ | .15^**^ | -.17^*^ | -.40^**^ |
|  | **4. Weight Concern** | 2.30 | 0.078 |  |  |  | - | .77^**^ | .90^**^ | .60^**^ | .95^**^ | -.16^**^ | .08 | .17^*^ |
|  | **5. Eating Concern** | 1.25 | 0.049 |  |  |  |  | - | .79^**^ | .58^**^ | .89^**^ | -.12 | .14^*^ | .27^**^ |
|  | **6. Shape Concern** | 2.65 | 0.089 |  |  |  |  |  | - | .60^**^ | .97^**^ | -.15^**^ | .12 | .26^**^ |
|  | **7. Restraint** | 1.72 | 0.078 |  |  |  |  |  |  | - | .91^**^ | -.03 | .03 | .02 |
|  | **8.**  **Eat. Path.** | 2.31 | 0.071 |  |  |  |  |  |  |  | - | -.13^*^ | .10 | .89^**^ |
|  | **9. Mature** | 0.082 | 0.132 |  |  |  |  |  |  |  |  | - | .17^**^ | .05 |
|  | **10. Neurotic** | 4.58 | 0.062 |  |  |  |  |  |  |  |  |  | - | .45^**^ |
|  | **11. Immature** | 3.44 | 0.050 |  |  |  |  |  |  |  |  |  |  | - |
|  | *Note.* ^*^significant to *p*<0.005; ^**^significant to *p*<0.001. Spearman rho (r_s_) correlations. Year-4 (Time 1; T1) intercorrelations for sample (n=1304). Psychological distress (Distress) was measured using the K10 scale; physical health related quality of life (PHQoL) and mental health quality of life (MHQoL) were measured using the Short Form 12; weight concerns, eating concerns, shape concerns, and restraint were measured using the Eating Disorder Examination Questionnaire (EDE-Q) and eating pathology (Eat. Path.) was the total/ global score across all subscales; mature, neurotic, and immature defence-styles were measured using the Defence-Style Questionnaire (DSQ). The current table highlights-cross sectional associations at T1. | | | | | | | | | | | | | |

**Table S4**

***Summary of Intercorrelations (Spearman’s rho), Means, and Standard Errors between T1 and T2 Defence-Styles subscales, Psychological Distress, HRQoL,& Eating Pathology (including subscales)***

|  |  |  |  | **Time 1 (Year 4)** | | | | | | | | | | |
| --- | --- | --- | --- | --- | --- | --- | --- | --- | --- | --- | --- | --- | --- | --- |
|  | ***Measures*** | **Mean** | **S.E** | **1** | **2** | **3** | **4** | **5** | **6** | **7** | **8** | **9** | **10** | **11** |
| **Time 2 (Year 9)** | **1. Distress** | 10.40 | 0.37 | .93^**^ | -.28^**^ | -.53^**^ | .92^**^ | .87^**^ | .93^**^ | .86^**^ | .93^**^ | -.15^**^ | .02 | -.03 |
|  | **2. PHQoL** | 50.19 | 0.34 | -.19^*^ | .30^**^ | .10 | -.23^**^ | -.18^**^ | -.18^**^ | -.18^*^ | -.23^**^ | .12 | -.07 | -.05 |
|  | **3. MHQoL** | 44.20 | 0.44 | -.29^**^ | .02 | .30^**^ | -.12 | -.18^*^ | -.21^**^ | -.07 | -.16^*^ | .11 | -.26^**^ | -.32^**^ |
|  | **4. WC** | 1.27 | 0.05 | .32^**^ | -.19^**^ | -.20^**^ | .52^**^ | .49^**^ | .52^**^ | .43^**^ | .56^**^ | -.10^*^ | .08 | .13 |
|  | **5. EC** | 1.71 | 0.03 | .31^**^ | -.10 | -.18^**^ | .43^**^ | .51^**^ | .46^**^ | .39^**^ | .51^**^ | -.13^*^ | .11 | .15^*^ |
|  | **6. SC** | 1.45 | 0.05 | .33^**^ | -.17^*^ | -.21^**^ | .47^**^ | .47^**^ | .51^**^ | .40^**^ | .53^**^ | -.14^**^ | .13 | .15^*^ |
|  | **7. Restraint** | 1.00 | 0.04 | .18^*^ | -.13 | -.10 | .36^**^ | .38^**^ | .37^**^ | .45^**^ | .44^**^ | .02 | .06 | .03 |
|  | **8. Eat. Path.** | 1.11 | 0.04 | .34^**^ | -.18^**^ | -.2^**^ | .49^**^ | .50^**^ | .52^**^ | .47^**^ | .56^**^ | .02 | .12 | .14^*^ |
|  | **9. Mature** | 5.37 | 0.05 | -.15^**^ | .12 | .11 | -.10^*^ | -.13^*^ | -.14^**^ | .02 | -.20^**^ | .60^**^ | .08 | -.14^**^ |
|  | **10. Neurotic** | 4.58 | 0.05 | .09 | -.07 | -.14^*^ | .00 | .08 | .03 | -.04 | -.00 | .25^**^ | .60^**^ | .30^**^ |
|  | **11. Immature** | 3.46 | 0.04 | .32^**^ | -.07 | -.24^**^ | .15^*^ | .23^**^ | .19^**^ | -.04 | .12 | -.14^**^ | .35^**^ | .62^**^ |
|  | **Mean** |  |  | 8.50 | 50.63 | 44.76 | 1.12 | 0.59 | 1.30 | 0.81 | 0.94 | 5.31 | 4.51 | 3.44 |
|  | **S.E** |  |  | 0.29 | 0.38 | 0.48 | 0.04 | 0.03 | 0.05 | 0.04 | 0.04 | 0.05 | 0.05 | 0.05 |
|  | *Note.* ^*^significant to *p*<0.005; ^**^significant to *p*<0.001. Spearman rho (r_s_) correlations. Year-4 (Time 1; T1) intercorrelations for participants (n=1304) are represented above the diagonal, and Intercorrelations for year-9 (Time 2; T2) participants (n=216) are presented below the diagonal. Means along with standard errors for T1 are presented in the vertical column, and mean and standard deviations for T2 are presented in the horizontal rows. Psychological distress (Distress) was measured using the K10 scale; physical health related quality of life (PHQoL) and mental health quality of life (MHQoL) were measured using the Short Form 12; weight concerns (WC), eating concerns (EC), shape concerns (SC), and restraint were measured using the Eating Disorder Examination Questionnaire (EDE-Q) and eating pathology (Eat. Path.) was the total/ global score across all subscales; mature, neurotic, and immature defence-styles were measured using the Defence-Style Questionnaire (DSQ). It should be noted that this table is a combined correlation matrix (as indicated in the Publication Manual of the American Psychological Association: 7th Edition) of associations *overtime*. | | | | | | | | | | | | | |

**Results**: A moderate significant negative association between T2 mature defence-style and T1 overall eating pathology, Spearman *r*_s_=-.20, p<.001, was found. There was a significant positive relationship between T1 immature defence-style and T2 overall eating pathology (*r*_s_=.14, p<.005). No other significant associations between T1 and T2 eating pathology and defence-style were found. As expected, there were significant associations between poorer HRQoL, greater psychological distress and greater eating pathology over time.

**Table S5**

***Influence of Psychological Distress, HRQoL, and Eating Pathology at T1 on Defence-Styles at T2***

| **Variables (T1)** | **B** | **SE** | **95% CI** | **sr^2^** | ***P value*** |
| --- | --- | --- | --- | --- | --- |
| **Dependent variable is Mature at T2** |  |  |  |  |  |
| Psychological Distress | -.003 | .007 | -.017,.012 | .009 | 0.7182 |
| PHQoL | .006 | .006 | -.006,.017 | .012 | 0.3312 |
| MHQoL* | -.012 | .004 | -.021,-.003 | .008 | 0.0060 |
| Weight Concern | .054 | .050 | -.045,.153 | .010 | 0.2820 |
| Eating Concern* | -.151 | .052 | -.254,-.049 | .019 | 0.0039 |
| Shape Concern* | -.106 | .051 | -.206,-.007 | .008 | 0.0358 |
| Restraint  T1 Mature** | .005  .145 | .038  .039 | -.070,.080  .069,.221 | .000  .028 | 0.8975  0.0002 |
| **Dependent variable is Immature at T2** |  |  |  |  |  |
| Psychological Distress | .010 | .006 | -.002,.022 | .005 | 0.0900 |
| PHQoL* | .009 | .005 | .000,.019 | .009 | 0.0470 |
| MHQoL | -.002 | .004 | -.009,.005 | .000 | 0.5704 |
| Weight Concern | .061 | .040 | -.018,.140 | .000 | 0.1323 |
| Eating Concern* | -.085 | .043 | -.168,-.001 | .013 | 0.0479 |
| Shape Concern | .035 | .041 | -.045,.115 | .000 | 0.3896 |
| Restraint**  T1 Immature | -.136  .054 | .031  .042 | -.197,-.076  -.029,.136 | .031  .003 | <0.0001  0.2013 |
| **Dependent variable is Neurotic at T2** |  |  |  |  |  |
| Psychological Distress*  T1 Neurotic | -.005  .071 | .008  .042 | -.021,-.011  -.012,.154 | .000  .003 | 0.0459  0.0953 |
| PHQoL | .008 | .006 | -.014,.015 | .000 | 0.2054 |
| MHQoL | -.003 | .005 | -.013,.006 | .000 | 0.4718 |
| Weight Concern | .061* | .054 | -.046,.167 | .001 | 0.2657 |
| Eating Concern | .041 | .056 | -.070,.152 | .000 | 0.4697 |
| Shape Concern | .040 | .055 | -.065,.148 | .000 | 0.4681 |
| Restraint** | - .151 | .041 | -.232,-.070 | .020 | 0.0003 |
| **Dependent variable is Overall Eating Pathology at T2** |  |  |  |  |  |
| Psychological Distress | .0005 | .0008 | -.01,.02 | .009 | 0.9508 |
| PHQoL** | -.027 | .006 | -.04,-.02 | .010 | <0.0001 |
| MHQoL** | -.035 | .004 | -.04,-.03 | .060 | <0.0001 |
| Weight Concern** | .201 | .057 | .09,.31 | .013 | 0.0005 |
| Eating Concern | .103 | .057 | .010,.217 | .003 | 0.0740 |
| Shape Concern | .031 | .057 | -.080,.143 | .000 | 0.5800 |
| Restraint* | .148 | .047 | .056,.240 | .009 | 0.0016 |
| Immaturity  T1 overall pathology | .085  .086 | .111  .093 | -.139,.309  -.097,.269 | .003  .312 | 0.4441  0.3582 |
| *Note.* ^*^significant to *p*<0.05; ^**^significant to *p*<0.001. All models controlled for year 4 age. | | | | | |
